# Custom-Made Compression Elastic Garments for Vascular Anomalies and Edematous Disorders: Objective and Subjective Outcomes in a Multi-Institutional Clinical Series

**DOI:** 10.3390/jcm15155819

**Published:** 2026-07-25

**Authors:** Sadanori Akita, Ai Morita, Yoshihisa Kawakami, Motoyuki Tamaki, Masanori Tamaki, Masaharu Tamaki

**Affiliations:** 1 Department of Plastic and Reconstructive Surgery, Tamaki Aozora Hospital, Tokushima 779-3125, Japan; 2 Department of Bioregulation and Pharmacological Medicine, Fukushima Medical University, Fukushima 960-1295, Japan; 3 Department of Plastic and Reconstructive Surgery, Fukuoka Children’s Hospital, Fukuoka 813-0017, Japan; 4 Department of Plastic and Reconstructive Surgery, Osaka Rosai Hospital, Sakai 591-8025, Japan; kawayossy@nifty.com; 5 Department of Diabetes, Metabolism, and Endocrinology, Tamaki Aozora Hospital, Tokushima 779-3125, Japan; tamaki@tamaki-aozora.ne.jp; 6 Department of Nephrology, Tamaki Aozora Hospital, Tokushima 779-3125, Japan; tamaki.masanori@tokushima-u.ac.jp (M.T.); tamaki_kf8@yahoo.co.jp (M.T.); 7 Department of Nephrology, Graduate School of Biomedical Sciences, Tokushima University, Tokushima 770-8503, Japan

**Keywords:** custom-made compression elastic garment, vascular anomaly, venous malformation, Klippel–Trénaunay syndrome, edema, lymphedema, compression therapy, conservative treatment, patient satisfaction, multi-institutional study

## Abstract

**Background/Objectives:** Compression therapy serves as first-line conservative management for low-flow vascular malformations and Klippel–Trénaunay syndrome (KTS). However, ready-made garments are frequently ill-fitting for patients with limb overgrowth, asymmetry, deformity, or heterogeneous body habitus. Custom-made compression elastic garments offer an individualized solution, yet systematic data across diverse clinical entities remain scarce. This study evaluated objective and subjective outcomes of custom-made compression garments across vascular anomalies and edematous disorders in a multi-institutional real-world setting. **Methods:** A retrospective observational case series was conducted between May 2023 and September 2025 at four institutions: a tertiary medical center, a pediatric specialty hospital, a corporate hospital, and a corporate clinic. Patients who received custom-made compression elastic garments for vascular anomalies, edema, varicose veins, post-traumatic or post-burn venous stasis, or postoperative conditions were included. Objective outcome (limb circumference change) and subjective outcome (patient satisfaction) were analyzed. **Results:** A total of 191 patients who received custom-made garments were described (117 females, 61.3%; 74 males, 38.7%; mean age 36.3 years; median 22 years; range 1–96 years). The age distribution was bimodal, with 91 patients (47.6%) aged 0–19 years and 50 patients (26.2%) aged ≥70 years. The tertiary center and pediatric hospital treated vascular anomaly patients exclusively (n = 102), while the corporate hospital and clinic primarily served adult and elderly edematous disorder patients (n = 88; one additional vascular anomaly case was managed at the corporate hospital). Quantitative paired outcome analysis was feasible in the subgroups with complete paired data: 20 vascular anomaly patients (objective outcome) and 20 edematous disorder patients (subjective outcome). In the vascular anomaly subgroup (n = 20), the affected limb showed a significantly greater circumference reduction at the ankle’s narrowest point (median −2 mm, IQR −4.2 to 0.0) compared with the unaffected contralateral limb (median +0.5 mm, IQR −0.2 to +1.0; Wilcoxon signed-rank test T = 4; *p* < 0.001; effect size r = 0.83). In the edematous disorder subgroup (n = 20), patient satisfaction scores improved significantly from a median of 3 (IQR 2.0–4.0) to 4 (IQR 3.0–6.0) after garment use (Wilcoxon signed-rank test T = 0; *p* < 0.001; effect size r = 0.83). Representative cases illustrated pediatric vascular anomaly (Case A, vascular malformation; Case C, KTS) and adult edematous disorder (Case B, chronic lower limb edema) presentations and outcomes. **Conclusions:** Custom-made compression elastic garments were feasible and well tolerated across a broad real-world spectrum of vascular anomalies and edematous disorders. In the analyzed subgroups, a measurable reduction in affected-limb circumference was observed in pediatric vascular anomaly patients, and patient satisfaction improved in adult and elderly edematous disorder patients. Because each subgroup was evaluated with only one outcome domain and no head-to-head comparison of measures was performed, these findings should be regarded as hypothesis-generating. We propose that outcome measures may need to be tailored to disease background and age, a hypothesis that warrants testing in prospective studies capturing both objective and subjective endpoints across all patient groups.

## 1. Introduction

Vascular anomalies encompass a heterogeneous group of congenital disorders classified by the International Society for the Study of Vascular Anomalies (ISSVA) into vascular tumors and vascular malformations [1]. Low-flow vascular malformations—including venous malformations (VMs), lymphatic malformations (LMs), and capillary malformations—result from structural abnormalities in vascular channels and commonly affect the extremities [2]. Klippel–Trénaunay syndrome (KTS) is a complex overgrowth disorder characterized by the clinical triad of capillary malformations, venous malformations, and soft tissue or bony hypertrophy of the extremities, and represents a prototype condition for conservative compression management [3,4]. Closely related PIK3CA-related overgrowth spectrum (PROS) disorders, including CLOVES syndrome, share overlapping pathophysiology and similar management challenges [5]. Beyond their structural impact, vascular malformations exert a substantial chronic burden on health-related quality of life (HRQoL): patients report significantly poorer scores than the general population across all SF-36 domains, with elevated rates of anxiety, depression, and bodily pain [6]. In KTS specifically, pain is reported by 63.4% of patients and is associated with diagnosed psychiatric comorbidities, particularly when lower-extremity or foot venous malformations are present [7]. Swelling, skin discoloration, and chronic and episodic pain are the most consistently reported symptoms in qualitative patient-reported outcome (PRO) studies of VM [8]. In these patients, limb enlargement and asymmetry are compounding problems that make individualized garment fitting essential.

Compression therapy is a cornerstone of conservative management for low-flow vascular malformations and KTS. Proposed mechanisms of benefit include mechanical reduction in venous pooling, relief of pain and heaviness, prevention of localized intravascular coagulation (LIC), and protection against minor trauma [9,10]. LIC is a consumptive coagulopathy—characterized by elevated D-dimer and decreased fibrinogen—that complicates venous malformations, particularly large-volume or deep-seated lesions, and compression therapy may attenuate its progression [11,12]. Beyond vascular anomalies, compression is well established in the management of post-thrombotic syndrome (PTS) [13,14], lymphedema [15,16], chronic venous insufficiency, post-traumatic and post-burn venous stasis, and postoperative venous congestion [17,18]. Consistent with this broad role, expert Delphi consensus on the physiotherapeutic management of venous ulcers identifies compression, together with exercise and multidisciplinary care, as a core non-pharmacological component of chronic venous disease management [19].

Despite widespread clinical use, the evidence base for compression therapy in vascular malformations is limited. A systematic review by Langbroek et al. identified only five observational studies (101 patients) from 565 candidate publications, concluding that while compression garments may yield symptomatic benefit, no high-quality prospective evidence exists to validate their use [9]. Consistent with this, recent GRADE-based clinical guidelines on compression therapy in venous diseases extend their scope to hemangiomas and vascular malformations yet are constrained by the limited high-quality evidence available for these entities [20]. Subsequent pharmacological alternatives—notably sirolimus—have demonstrated clinical benefit in patients with venous and lymphatic malformations [21,22], yet compression therapy retains its role as a low-cost, non-invasive first-line adjunct.

A critical practical challenge is that ready-made compression garments frequently fail to fit patients with vascular anomalies due to limb overgrowth, anatomical asymmetry, or postoperative deformity [3,23]. Custom-made compression elastic garments address this by providing individualized fit. A multi-institutional prospective study by Noguchi et al. in patients with KTS and VM demonstrated that 30 mmHg custom-made garments worn for 26 weeks produced significant improvement in affected-to-unaffected limb circumference ratios at the ankle and tibial tuberosity, without major adverse events [23]. Elastic force declined approximately 50% at 26 weeks, suggesting replacement at least every six months. However, data extending beyond KTS and VM—encompassing adult edematous disorders, chronic venous insufficiency, post-burn and post-traumatic conditions, and postoperative states—are lacking.

Evaluating conservative treatments such as compression requires attention to how outcomes are measured. Objective anthropometric endpoints (e.g., limb circumference or volume) capture the mechanical effect of compression, whereas patient-centered instruments capture the experience and impact of care. Patient-reported outcome measures (PROMs) quantify the patient’s own assessment of symptoms, function, and health status, while patient-reported experience measures (PREMs) capture the patient’s experience of the care process, including comfort, tolerability, and satisfaction with the device. In heterogeneous, chronic, and often incurable conditions such as vascular anomalies and edematous disorders, objective and patient-centered measures are complementary rather than interchangeable, and the most informative endpoint may depend on the underlying disease and the goals of treatment. This consideration directly motivates our parallel evaluation of an objective endpoint (limb circumference) and a subjective, satisfaction-based endpoint in the present study.

This multi-institutional retrospective case series was designed to address this gap by describing 191 consecutive patients who received custom-made compression elastic garments across four institutions with distinct patient populations, and by quantifying, within the subgroups for which complete paired data were available, an objective outcome (limb circumference) and a subjective outcome (patient satisfaction). We explored whether the outcome domain that could be meaningfully captured differed according to disease background and patient age group, recognizing that this real-world dataset is descriptive and hypothesis-generating rather than confirmatory.

## 2. Materials and Methods

### 2.1. Study Design and Setting

This was a multi-institutional retrospective observational case series conducted between May 2023 and September 2025, reported in accordance with the STROBE (Strengthening the Reporting of Observational Studies in Epidemiology) guidelines [24]. The study was performed at four institutions in Japan: (1) a tertiary academic medical center specializing in vascular anomalies; (2) a pediatric specialty hospital with a dedicated vascular anomalies program; (3) Tamaki Aozora Hospital—a corporate hospital serving adult and elderly patients; and (4) Aizumi Tamaki Aozora Clinic—an affiliated corporate outpatient clinic. These four institutions were deliberately selected because of their complementary structural differences: together they span the full range of care settings in which custom-made compression garments are prescribed in Japan, from tertiary academic and pediatric specialty practice to community-based general hospital and outpatient care. They share a common custom-garment supplier and referral network, which allowed consistent garment fabrication and measurement practices while capturing markedly different patient populations (pediatric vascular anomalies versus adult and elderly edematous disorders). Ethical approval and consent procedures are detailed in Section 2.6.

### 2.2. Patient Eligibility

**Figure 1:**
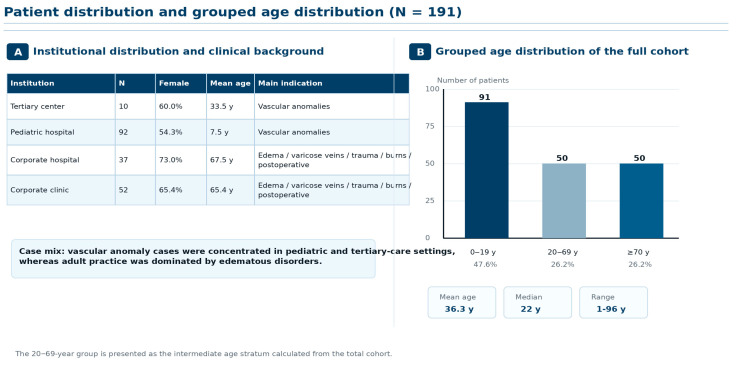
Patient distribution, institutional case mix, and grouped age distribution across the four participating institutions. (**A**) Institutional case numbers, female proportion, mean age, and principal clinical indications are summarized. Vascular anomaly cases were concentrated in the tertiary medical center and pediatric specialty hospital, whereas adult and elderly edematous disorder cases predominated in the corporate hospital and clinic. (**B**) Grouped age distribution of the full cohort. The distribution was bimodal, with a primary pediatric group aged 0–19 years (n = 91, 47.6%) and an older adult group aged ≥70 years (n = 50, 26.2%). The overall mean age was 36.3 years, the median age was 22 years, and the range was 1–96 years.

This study used consecutive (non-probability) sampling: all consecutive patients who received at least one pair of custom-made compression elastic garments during the study period were eligible for inclusion. Indications for garment prescription included: (1) vascular anomalies (VMs, KTS, LMs, combined malformations, and other ISSVA-classified entities); (2) primary or secondary lymphedema; (3) chronic venous insufficiency and varicose veins; (4) post-traumatic or post-burn venous stasis; (5) postoperative venous stasis following skin grafting or flap reconstruction; and (6) other edematous conditions requiring individualized compression. Patients were excluded if follow-up data were insufficient to permit any outcome assessment. The full descriptive cohort comprised 191 patients; the analytic samples for the paired quantitative outcomes comprised the subgroups with complete paired data (n = 20 for the objective outcome and n = 20 for the subjective outcome; see Section 2.5 and Figure 1). No formal a priori sample-size calculation was performed, as this was a retrospective analysis of all available real-world cases.

### 2.3. Custom-Made Compression Elastic Garments

All garments were individually fabricated by a licensed compression garment manufacturer based on standardized circumferential measurements taken by trained clinical staff. The measurement points included the ankle (narrowest point), tibial tuberosity level, mid-calf, and mid-thigh, following the protocol described by Noguchi et al. [23]. Circumferential tape measurement at these standardized landmarks has been shown to have high intra- and inter-rater reliability for the assessment of lower-limb volume change in clinical practice [25]. Compression class (pressure range) was determined by the treating clinician based on clinical indication, patient tolerance, and skin condition. Garments were provided for affected limb segments (lower extremity, upper extremity, or truncal regions) as clinically indicated.

### 2.4. Outcome Measures

Two primary outcome domains were evaluated. Objective outcome: Limb circumference change was measured at standardized anatomical landmarks before and after garment use. At the tertiary medical center and pediatric specialty hospital, the ratio of affected-to-contralateral unaffected limb circumference was calculated at multiple sites following the method of Noguchi et al. [23]. Improvement was defined as a reduction in this ratio at ≥1 measurement site. Subjective outcome: Patient-reported satisfaction was assessed using a structured questionnaire administered at follow-up visits, evaluating overall satisfaction, perceived symptom relief (pain, heaviness, swelling), and ease of donning and doffing. The selection of these symptom domains was informed by qualitative PRO research demonstrating that swelling, pain, and functional limitation are the most consistently reported patient concerns in vascular and edematous lower-limb disorders [8]. Satisfaction improvement was defined as a categorical improvement in the global satisfaction rating from baseline to follow-up. Additional data collected included demographics, underlying diagnosis (ISSVA-categorized for vascular anomaly cases; primary clinical indication for others), affected region, garment type, compression class, wearing adherence (self-reported), skin complications, and need for replacement or refitting.

Representative cases were purposively selected to illustrate the clinical and age-spectrum diversity of the cohort—specifically, pediatric vascular anomaly (a lower-extremity venous malformation and a case of KTS) and elderly edematous disorder (chronic lower-limb edema)—rather than to represent typical or best-case responders. All clinical photographs were obtained during routine clinical care using standardized positioning, and written informed consent for the publication of identifiable clinical images was obtained from all depicted patients or their legal guardians.

### 2.5. Statistical Analysis

Descriptive statistics were used to summarize demographics and clinical characteristics. Categorical variables are expressed as frequencies and percentages; continuous variables as mean ± SD or median (interquartile range [IQR]) as appropriate. Of the 191 patients in the full descriptive cohort, quantitative paired outcome analysis was restricted to patients with complete paired data at the identical assessment: 20 patients with vascular anomalies had both pre- and post-garment bilateral circumference measurements at the ankle’s narrowest point (objective outcome), and 20 patients with edematous disorders had both pre- and post-garment satisfaction scores (subjective outcome). The remaining patients were included in the descriptive characterization of the cohort but did not contribute to the paired analyses because standardized paired data were not uniformly recorded in this retrospective real-world setting (Figure 1). Normality of continuous outcome variables was assessed using the Shapiro–Wilk test. As the distribution of both outcome variables was non-normal, non-parametric methods were used throughout. For the objective outcome, the Wilcoxon signed-rank test was used to compare the change in circumference at the ankle’s narrowest point between the affected and unaffected limbs (paired analysis, n = 20). For the subjective outcome, the Wilcoxon signed-rank test was used to compare patient satisfaction scores before and after garment use (paired analysis, n = 20). Effect size for non-parametric paired tests was calculated as r = |Z|/√N, where Z is the standardized Wilcoxon test statistic and N is the total number of observations contributing to the test, with conventional thresholds of r = 0.1 (small), 0.3 (medium), and 0.5 (large). To quantify the precision of the estimates given the small analytic samples, we computed nonparametric bootstrap 95% confidence intervals (CIs; 10,000 resamples with replacement) for both the effect size r and the median paired difference for each outcome. A two-sided *p* < 0.05 was considered statistically significant. All analyses were performed using Python (version 3.11, 2023 release; Python Software Foundation, Wilmington, DE, USA) with the SciPy library (version 1.11, 2023).

### 2.6. Ethical Considerations

The study was conducted in accordance with the Declaration of Helsinki. The study protocol was reviewed and approved by the Institutional Review Board of Tamaki Aozora Hospital (approval no. 9; date of approval: 11 May 2026), which served as the central ethics committee for this multi-institutional retrospective study. In accordance with institutional policies governing retrospective, non-interventional, records-based research, the participating institutions accepted this central approval. As a purely observational retrospective case series, the study did not require clinical trial registration. Written or waived informed consent for the use of clinical data was obtained in accordance with institutional policies, and written informed consent for the publication of identifiable clinical photographs was obtained from all depicted patients or their legal guardians.

## 3. Results

### 3.1. Overall Patient Characteristics

A total of 191 patients who received custom-made compression elastic garments between May 2023 and September 2025 were included. The cohort comprised 117 females (61.3%) and 74 males (38.7%). The mean age was 36.3 years and the median age was 22 years (range 1–96 years). The age distribution was bimodal: 91 patients (47.6%) were aged 0–19 years, and 50 patients (26.2%) were aged ≥70 years. The broad age range underscored the diversity of the study population (Figure 1).

### 3.2. Institution-Specific Demographics and Case Mix

The clinical characteristics differed substantially among institutions, reflecting distinct patient populations and referral patterns (Table 1). The pediatric specialty hospital contributed the largest number of cases (n = 92; 48.2%), with a mean age of 7.5 years, median age of 6 years, female proportion of 54.3%, and 95.7% of patients aged <20 years. The tertiary medical center included 10 patients (mean age 33.5 years; median age 30 years; female 60.0%), all with vascular anomalies. The corporate hospital (n = 37; mean age 67.5 years; median age 74 years; female 73.0%) and corporate clinic (n = 52; mean age 65.4 years; median age 72 years; female 65.4%) primarily treated adult and elderly patients with edematous disorders. Combined, the tertiary center and pediatric hospital provided 102 vascular anomaly patients. The corporate sites provided 88 edematous disorder patients and one additional vascular anomaly case at the corporate hospital; thus, the full cohort comprised 103 vascular anomaly cases (53.9%) and 88 edematous disorder cases (46.1%).

### 3.3. Objective Outcomes

**Figure 2:**
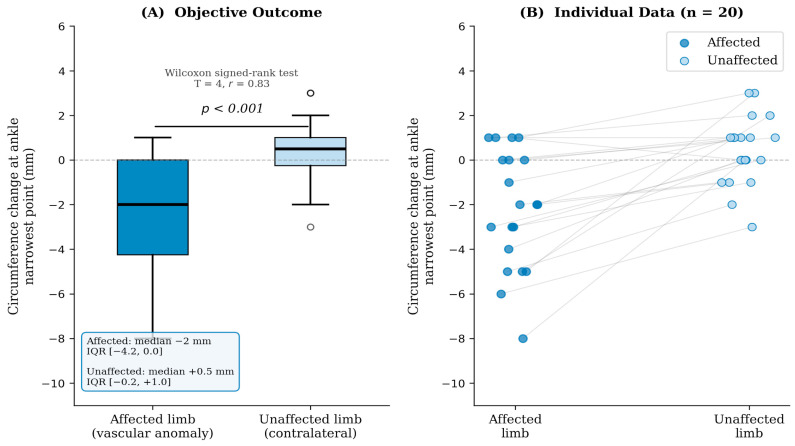
Objective outcome: circumference change at the ankle’s narrowest point in patients with vascular anomalies (n = 20). (**A**) Box-and-whisker plots comparing circumference change in the affected limb (blue) and the unaffected contralateral limb (light blue). The affected limb showed a median decrease of 2 mm (IQR −4.2 to 0.0), whereas the unaffected limb showed a median increase of +0.5 mm (IQR −0.2 to +1.0). Wilcoxon signed-rank test: T = 4, *p* < 0.001; effect size r = 0.83 (bootstrap 95% CI 0.71–0.89). (**B**) Individual paired data. Each point represents one patient; gray lines connect paired affected and unaffected measurements. A negative value indicates circumference reduction (improvement).

Circumference changes at the ankle’s narrowest point were assessed in 20 patients with vascular anomalies who had complete bilateral measurements (affected and contralateral unaffected limb) available before and after custom-made compression garment use. Data were non-normally distributed (Shapiro–Wilk test: affected limb W = 0.856, *p* = 0.007; unaffected limb W = 0.856, *p* = 0.007), and a Wilcoxon signed-rank test was therefore used. The affected limb showed a median circumference change of −2 mm (IQR −4.2 to 0.0), indicating a reduction in limb circumference. In contrast, the unaffected contralateral limb showed a median change of +0.5 mm (IQR −0.2 to +1.0), consistent with natural limb-circumference variation over time. The difference between affected and unaffected limb changes was statistically significant (Wilcoxon signed-rank test: T = 4; *p* < 0.001), with a large effect size (r = 0.83; bootstrap 95% CI 0.71–0.89). The median paired difference between the affected and unaffected limbs was −2 mm (bootstrap 95% CI −3 to −1 mm). In this subgroup, limb circumference at the ankle was therefore a feasible and measurable objective endpoint that discriminated the affected limb from the unaffected contralateral limb during custom-made compression garment use. Two caveats apply to its interpretation. First, because this was an uncontrolled retrospective comparison without an untreated reference group, these data describe an association and should not be interpreted as establishing a causal treatment effect; regression to the mean, spontaneous clinical fluctuation, and concurrent management could contribute to the observed difference. Second, no minimal clinically important difference (MCID) has been established for ankle circumference in vascular malformations, and the clinical importance of a 2 mm median difference is therefore uncertain and was not linked in this study to symptoms, function, or long-term disease progression. Results are summarized in Table 2 and Figure 2.

### 3.4. Subjective Outcomes

**Figure 3:**
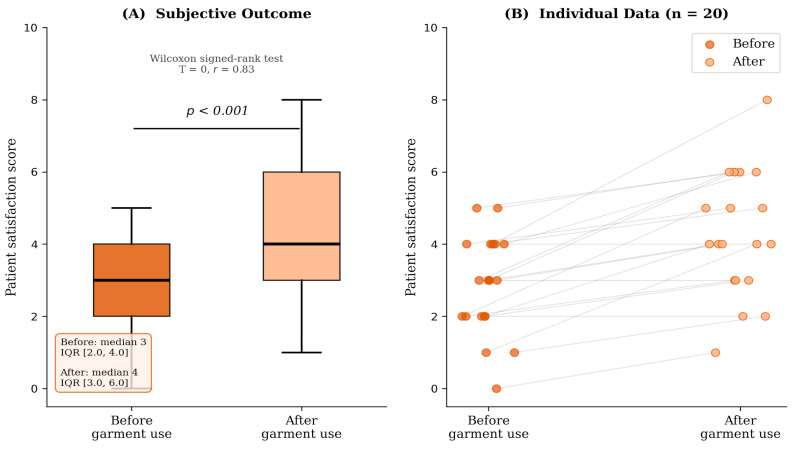
Subjective outcome: patient satisfaction scores before and after custom-made compression garment use in the edematous disorder group (n = 20). (**A**) Box-and-whisker plots comparing satisfaction scores before (dark orange) and after (light orange) garment use. The median score increased from 3 (IQR 2.0–4.0) to 4 (IQR 3.0–6.0). Wilcoxon signed-rank test: T = 0, *p* < 0.001; effect size r = 0.83 (bootstrap 95% CI 0.75–0.91). (**B**) Individual paired data. Each point represents one patient; gray lines connect paired pre- and post-measurements for the same patient. Higher scores indicate greater satisfaction.

Patient-reported satisfaction scores were analyzed in 20 patients with edematous disorders who had complete paired pre- and post-garment satisfaction data. The score distribution was non-normal (Shapiro–Wilk test: before W = 0.852, *p* = 0.006; after W = 0.852, *p* = 0.006), and a Wilcoxon signed-rank test was used. The median satisfaction score increased significantly from 3 (IQR 2.0–4.0) before garment use to 4 (IQR 3.0–6.0) after garment use (Wilcoxon signed-rank test: T = 0; *p* < 0.001), with a large effect size (r = 0.83; bootstrap 95% CI 0.75–0.91). The median paired improvement was +1 point (bootstrap 95% CI +1 to +2). Notably, 19 of 20 patients (95.0%) showed no decrease in satisfaction score, and 17 of 20 patients (85.0%) demonstrated a categorical score improvement. Because the satisfaction instrument was not formally validated and no MCID has been established for it, the clinical magnitude of a one-point median increase is uncertain. These improvements were documented across a heterogeneous population including chronic venous edema, varicose veins, post-traumatic venous stasis, post-burn conditions, and postoperative venous congestion. Results are summarized in Table 3 and Figure 3.

### 3.5. Representative Cases

The following representative cases are provided for illustrative and educational purposes only. They were purposively selected to depict the clinical and age-spectrum diversity of the cohort and do not constitute systematically analyzed data or evidence of treatment efficacy; their value is illustrative rather than evidentiary.

**Figure 4:**
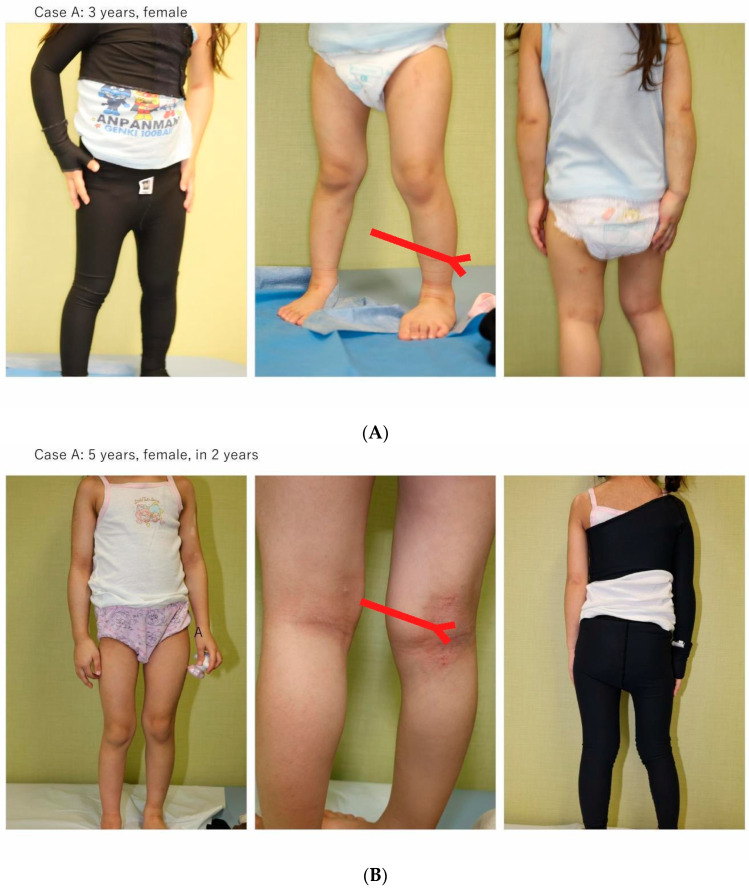
(**A**). Representative pediatric case (Case A) with left lower-extremity venous malformation at initial presentation (3 years). Bilateral lower-extremity asymmetry and the custom-made compression garment are shown. Arrows indicate the affected region (the enlarged left lower limb). (**B**). Same patient (Case A) at 5 years of age, after 2 years of continuous custom-made compression garment use. Stabilized lower-extremity asymmetry and garment adaptation to growth are demonstrated.

**Case A: Vascular malformation of the lower extremity (pediatric).** A 3-year-old girl presented with left lower extremity venous malformation involving the thigh, calf, and ankle, resulting in limb asymmetry (Figure 4A). Custom-made lower-extremity compression garments were introduced early to prevent progressive limb deformity. At 5 years of age (two years of garment use), objective circumference ratios had stabilized and the degree of left lower limb swelling was attenuated compared with expected natural progression (Figure 4B). The garments were well tolerated without skin complications.

**Figure 5:**
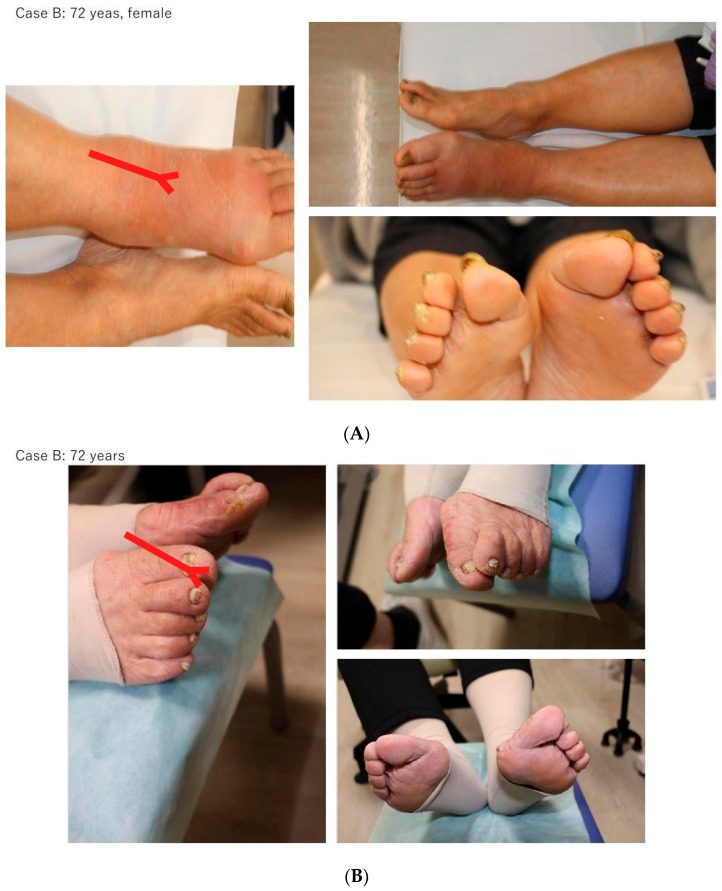

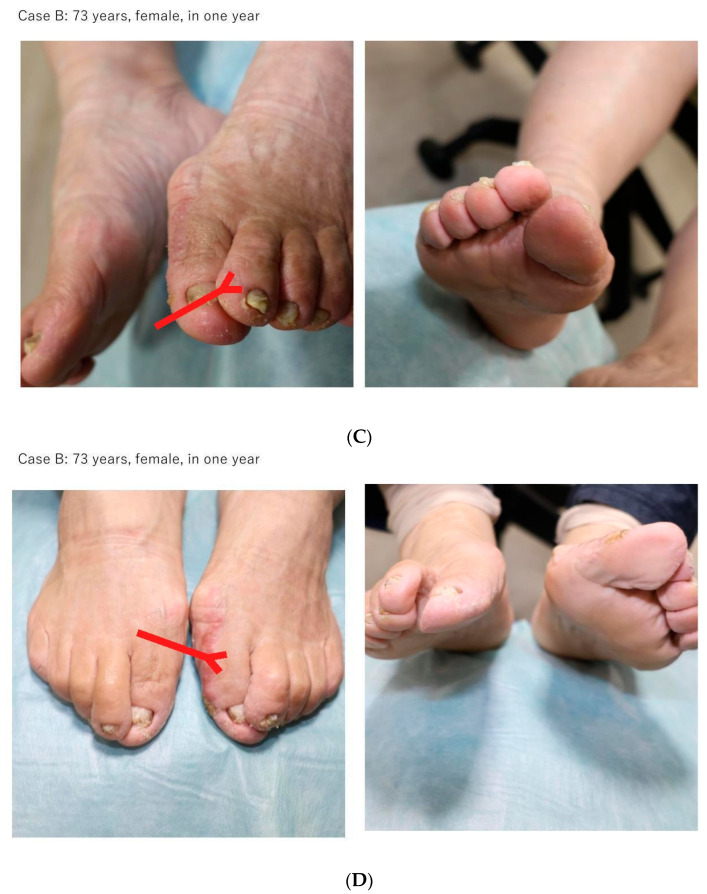
(**A**). Case B-1 (72-year-old woman): chronic lower-limb edema and venous insufficiency-related skin changes at baseline. Arrows indicate the affected edematous region. (**B**). Case B-1: Detail views showing foot deformity and the anatomically accommodating fit of the custom-made compression garment. (**C**). Case B-2 (73-year-old woman): chronic lower-limb edema, digital contractures, and nail changes at baseline. (**D**). Case B-2: One-year follow-up demonstrating maintained improvement with custom-made compression garment use.

**Case B: Chronic lower limb edema in elderly female patients. Case B-1:** A 72-year-old woman presented with bilateral lower limb edema with skin erythema, hyperpigmentation, and toe deformities consistent with chronic venous insufficiency (Figure 5A,B). Custom-made lower limb compression garments were prescribed. At one year of follow-up, patient satisfaction had significantly improved, with reported reductions in leg heaviness and swelling. **Case B-2:** A 73-year-old woman with chronic lower limb edema complicated by digital contractures and skin changes (Figure 5C,D) similarly reported sustained subjective satisfaction with custom-made compression after one year of use, highlighting the applicability of individualized garments in elderly patients with complex lower extremity morphology.

**Figure 6:**
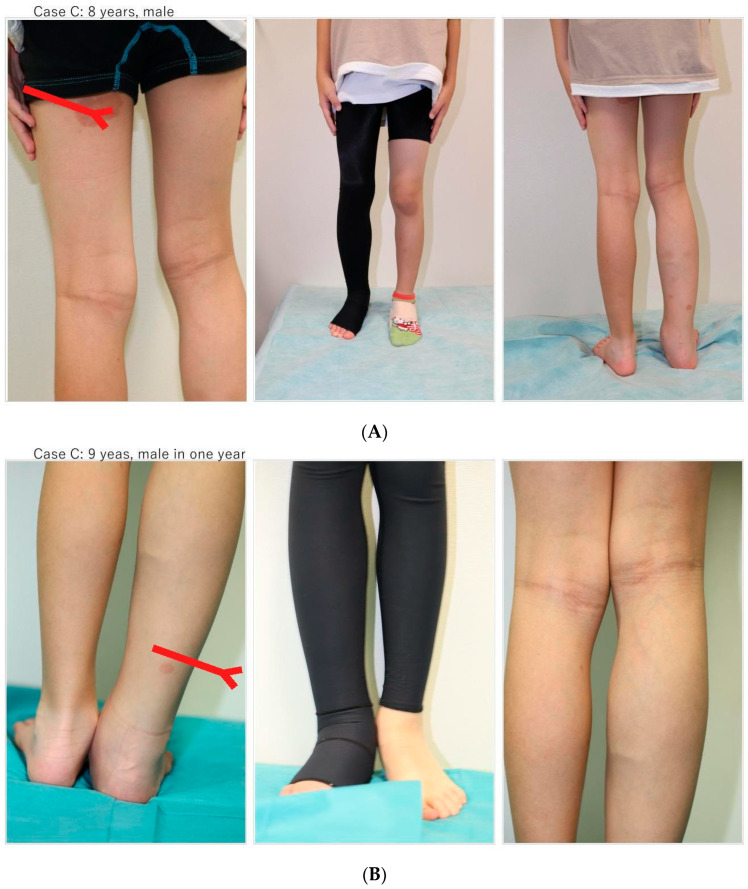
(**A**). Case C: An 8-year-old boy with right lower-extremity vascular malformation/Klippel–Trénaunay syndrome at initial presentation. Capillary malformation and limb asymmetry are shown; arrows indicate the capillary malformation (port-wine stain) on the posterior thigh. (**B**). Case C at 9 years of age, after 1 year of custom-made compression garment use. Improved lower-leg symmetry and reduction in distal swelling are evident.

**Case C: Vascular malformation of the lower extremity (pediatric, KTS).** An 8-year-old boy presented with unilateral right lower limb vascular malformation (capillary malformation visible on the thigh) with associated limb swelling (Figure 6A). A custom-made long lower-extremity compression garment was introduced. One year later, at 9 years of age, objective circumference improvement was observed in the right lower extremity, with maintained symmetry relative to the contralateral limb (Figure 6B). The garment was worn consistently without reported adverse events.

## 4. Discussion

The present multi-institutional case series described 191 patients treated with custom-made compression elastic garments across four institutions over 28 months and quantified paired outcomes in two subgroups of 20 patients each. It shows that custom-made compression garments can be prescribed and evaluated across a wide real-world spectrum of vascular anomalies and edematous disorders, and that a measurable objective change (limb circumference) was demonstrable in the vascular anomaly subgroup while a subjective improvement (patient satisfaction) was demonstrable in the edematous disorder subgroup. Importantly, each subgroup was evaluated with only one outcome domain, and we did not compare the two measures head-to-head within any group. We therefore do not claim that limb circumference is the superior or preferred endpoint in vascular anomalies, nor that satisfaction is superior in edematous disorders; both objective and patient-centered measures are likely to be informative in both populations. This restraint is essential because disease category, patient age, treating institution, clinical expertise, and the outcome-assessment methodology were mutually and inextricably confounded in this dataset: vascular anomaly patients were young and assessed by circumference at the tertiary and pediatric centers, whereas edematous disorder patients were older and assessed by satisfaction at the community sites. Because the two outcome domains were never evaluated simultaneously within the same population, the study cannot establish which endpoint is preferable in which group. Rather, our observations generate the hypothesis that the most feasible and informative outcome domain may vary with disease background and age—a hypothesis with potential implications for clinical trial design and individualized patient counseling that requires prospective testing.

The evidence base for compression therapy in low-flow vascular malformations and KTS remains critically limited. Langbroek et al.’s systematic review found only five observational studies totaling 101 patients from 565 publications; benefits included reduction in LIC, symptom improvement, and edema reduction, but standardized outcomes and harms data were consistently absent [9]. In terms of descriptive enrollment, the present study contributes a real-world cohort of 191 patients treated with custom-made garments—larger than the entire body of evidence reviewed by Langbroek et al.—and extends the clinical spectrum beyond classically studied entities. We emphasize, however, that this numerical advantage applies only to descriptive enrollment: the quantitative conclusions rest on only 20 paired observations per endpoint, so the study extends the descriptive clinical experience and generates hypotheses rather than substantially expanding the quantitative evidence base. Our objective outcome data—showing a median ankle circumference reduction of 2 mm in the affected limb (Wilcoxon signed-rank test, T = 4, *p* < 0.001, effect size r = 0.83; bootstrap 95% CI 0.71–0.89) with a clearly contrasting near-zero change in the unaffected contralateral limb—are consistent with, but do not by themselves establish, a site-specific mechanical effect of custom-made compression. In the absence of an untreated comparator and a longitudinal within-limb baseline, alternative explanations such as regression to the mean, spontaneous clinical fluctuation, and concurrent management cannot be excluded, and the observed affected-versus-unaffected difference should be interpreted as an association rather than as proof of a treatment effect. This pattern is nonetheless directionally consistent with the prospective data of Noguchi et al. [23], who demonstrated significant circumference ratio improvement at the ankle and tibial tuberosity levels following six months of 30 mmHg custom-made garment use in KTS with VM.

The pathophysiological rationale for objective circumference benefit in vascular anomaly patients is well supported. Venous pooling drives progressive limb enlargement in VMs and KTS, and the hydrodynamic forces involved in early childhood are particularly relevant to long-term morphological outcome. LIC, a consumptive coagulopathy driven by stasis in large-volume VMs, may be mechanically attenuated by external compression [11,12]. Early institution of compression in pediatric patients—as illustrated by Case A and Case C—may limit the cumulative anatomical deformity that accrues over years of untreated venous hypertension, a hypothesis that requires prospective validation. Importantly, the chronic pain burden in KTS, reported in over 60% of patients and associated with lower-extremity and foot venous malformations [7], provides an additional rationale for early conservative intervention beyond mechanical containment alone. Within the contemporary multidisciplinary management framework for vascular anomalies, conservative and medical measures remain the first-line approach, with interventional treatment reserved for cases in which they prove insufficient [26]. Sirolimus has demonstrated volume reduction in lymphatic malformations and symptom improvement in combined malformations [21,22], but compression therapy retains complementary value as a low-cost, non-invasive, continuous-use adjunct that does not require systemic drug tolerance.

For adult and elderly patients with edematous disorders—as exemplified by Case B—the clinical rationale and expected outcomes differ fundamentally from vascular anomaly management. In chronic venous insufficiency, PTS, lymphedema, post-burn, and postoperative edema, the goals are symptomatic relief, maintenance of daily function, and quality of life preservation [13,14,15,16]. The relevance of patient-reported outcomes (PROs) in this domain is well established: across all SF-36 subscales, vascular and edematous disorders impair HRQoL well below general-population norms, with bodily pain and role-physical functioning being particularly affected [6], and the symptoms most consistently reported by patients—swelling, pain, and functional limitation—map directly onto the satisfaction domains evaluated in our questionnaire [8]. Our subjective outcome data demonstrated a statistically significant and clinically meaningful improvement in patient satisfaction scores (median increase from 3 to 4; Wilcoxon signed-rank test, T = 0, *p* < 0.001, effect size r = 0.83), with 85.0% of patients (17/20) showing a categorical score improvement. A large effect size for a patient-reported outcome in a heterogeneous real-world population suggests that the benefit of custom-made fit was perceived consistently across diverse diagnostic subgroups. Patient-reported outcomes are valuable in these populations, consistent with evidence from cancer-related lymphedema trials showing improvements in patient-reported outcomes with compression regardless of whether volume reduction is complete [15,17]. We emphasize, however, that this does not imply that objective measures are less relevant in adults: limb circumference or volumetric assessment can be equally meaningful in adult edematous disorders, and the predominance of satisfaction data at the community sites reflects real-world data-availability patterns rather than any evidence that circumference is uninformative in this group. An important caveat is that lower-limb edema in adults is frequently multifactorial (cardiac, renal, hepatic, venous, lymphatic, and drug-induced contributions), and concurrent changes in medications such as diuretics or antihypertensives could contribute to symptomatic improvement independently of the garment. Because concomitant medications and their changes over the observation period were not systematically captured, the observed satisfaction improvement cannot be attributed to compression alone. Custom-made garments may nonetheless offer better fit and comfort than ready-made alternatives in elderly patients with complex lower-extremity morphology—as illustrated by both patients in Case B, both of whom had atypical foot and toe architecture that would preclude standard commercial sizing.

A distinguishing feature of the present study is its deliberate multi-institutional design spanning tertiary academic, pediatric, and community practice settings. This breadth reflects the genuine heterogeneity of clinical contexts in which custom-made compression garments are prescribed and ensures external validity across care environments. The extreme age range (1–96 years) and bimodal distribution underline that ready-made compression garments are systematically unsuitable at both ends of the age spectrum: pediatric vascular anomaly patients present anatomically atypical limb proportions, while elderly patients frequently have lower limb deformities, reduced skin integrity, and functional limitations precluding standard commercial sizing [3,23]. Ready-made garments in both populations risk inadequate compression, skin trauma, or non-adherence.

Garment non-adherence is a recognized challenge in the burn rehabilitation literature, with Crofton et al. identifying six thematic barriers including sensory discomfort, psychological burden, and functional impact [18]. The high satisfaction rates in our adult edematous disorder group may partly reflect superior garment comfort and fit with custom-made devices compared with off-the-shelf products, potentially reducing these adherence barriers. Prospective assessment of adherence and its determinants in custom-made versus ready-made compression garment users is an important direction for future research.

Several limitations of this study must be acknowledged. First, the retrospective observational design precludes causal inference, and the absence of an untreated comparator group limits attribution of outcomes to garment use. Second, although 191 patients were described, the quantitative outcome analyses were restricted to two subgroups of 20 patients each who had complete paired data—together only 40 patients, or 20.9% of the enrolled population. These subgroups were not randomly selected but were defined by data availability, which introduces a substantial risk of selection and attrition bias; patients with complete paired measurements may differ systematically from those without, and the analytic samples may not represent the full cohort. The study therefore functions primarily as a descriptive case series rather than a comparative effectiveness investigation, and the findings should be interpreted as hypothesis-generating rather than confirmatory. Although we report bootstrap 95% confidence intervals to quantify precision, estimates derived from only 20 pairs remain sensitive to individual observations and may be unstable, so the confidence intervals should themselves be regarded as approximate. Third, the heterogeneity of disease indications, age strata, and institutional practices across four institutions restricts direct inter-group comparison, and disease and institution were closely confounded (vascular anomalies at the tertiary and pediatric sites; edematous disorders at the community sites). Moreover, both broad groups remained internally heterogeneous in pathophysiology, natural history, and expected treatment response—the vascular anomaly group spanning venous, lymphatic, and combined malformations and KTS, and the edematous group spanning chronic venous insufficiency, primary and secondary lymphedema, and post-traumatic, post-burn, and postoperative edema—which limits internal validity and the interpretation of pooled estimates. The small paired-data subgroups (n = 20 each) precluded meaningful prespecified disease-specific subgroup analysis, which would have provided considerably more informative evidence and should be a priority for adequately powered prospective studies. Fourth, outcome measurement was not fully standardized across institutions: circumference-based assessments were performed at the vascular anomaly centers, while satisfaction-based assessments predominated at the community sites, reflecting real-world clinical priorities rather than a prospectively planned differentiation; consequently, each subgroup was evaluated with only a single outcome domain, precluding any within-group comparison of the relative merits of objective versus subjective endpoints. Fifth, the satisfaction instrument was a structured but non-validated questionnaire, and the minimal clinically important difference for its scores has not been established. Sixth, in adult patients with frequently multifactorial edema, concomitant medications and their changes (e.g., diuretics, antihypertensives) and other co-interventions—including venous procedures, physiotherapy, and manual lymphatic drainage—were not systematically recorded and may have contributed to the observed symptomatic improvement independently of the garment. Seventh, treatment adherence was assessed only by self-report at follow-up visits and was not objectively verified (e.g., by wear-time monitoring or standardized diaries); self-reported adherence is subject to recall and social-desirability bias and was not uniformly documented. Eighth, detailed data on compression class, wearing hours, adverse events, garment replacement intervals, and cost were not uniformly captured, and the small tertiary-center sample (n = 10) further limits subgroup statistical power. These limitations are characteristic of real-world observational data and should be addressed in future prospective studies.

Future research should prioritize prospective multicenter cohort studies or randomized controlled trials with disease-stratified enrollment, standardized and predetermined compression protocols, predefined follow-up intervals, and systematic adverse-event monitoring. Critically, both objective volumetric assessment (limb circumference or volume) and validated patient-reported outcome and experience measures (PROMs and PREMs) should be collected in every participant across all disease and age groups, so that the relative informativeness of each measure can be compared directly within the same population rather than inferred from the endpoint that happened to be available. Prespecified disease-specific subgroup analyses should be planned and adequately powered. Studies should also systematically capture concomitant medications and co-interventions and their changes over time, and should incorporate objective measurement of garment adherence (e.g., wear-time monitoring) rather than self-report alone. Finally, incorporation of validated health-related quality-of-life instruments, functional mobility assessment, cost-effectiveness analysis, and long-term adherence data would substantially strengthen the evidence base. The UMIN-registered prospective study by Noguchi et al. (UMIN000038282) [27] provides a methodological template that could be extended to broader disease entities.

## 5. Conclusions

The aim of this study was to describe the real-world use of custom-made compression elastic garments across a broad spectrum of vascular anomalies and edematous disorders and to evaluate objective and subjective outcomes where feasible. In answer to this aim, we found that custom-made garments were prescribed and tolerated across a wide range of diagnoses and ages (1–96 years); that, in the vascular anomaly subgroup with paired data, affected-limb circumference at the ankle was reduced relative to the unaffected limb; and that, in the edematous disorder subgroup with paired data, patient satisfaction improved after garment use. These are uncontrolled, single-domain observations in small subgroups drawn from a larger descriptive cohort, and they do not establish that either measure is superior to the other in any group. Their principal value is to generate the hypothesis that the most feasible and informative outcome domain may depend on disease background and age. Prospective studies that capture both objective and patient-reported endpoints in all patient groups, with standardized protocols and objective adherence monitoring, are needed to test this hypothesis and to define the true clinical benefit of custom-made compression therapy.

## Figures and Tables

**Table 1:** Institution-specific demographics and clinical indications of patients receiving custom-made compression elastic garments.

Institution	n (%)	Female, n (%)	Age, Mean/Median (y)	Principal Clinical Indications/Case Mix
Tertiary medical center	10 (5.2)	6 (60.0)	33.5/30	Vascular anomalies (10/10, 100%)
Pediatric specialty hospital	92 (48.2)	50 (54.3)	7.5/6	Vascular anomalies (92/92, 100%); 88/92 (95.7%) aged <20 y
Corporate hospital	37 (19.4)	27 (73.0)	67.5/74	Edematous disorders, varicose veins, post-traumatic/burn, or postoperative (36/37, 97.3%); one vascular anomaly case
Corporate clinic	52 (27.2)	34 (65.4)	65.4/72	Edematous disorders, varicose veins, post-traumatic/burn, or postoperative (52/52, 100%)
Total	191 (100)	117 (61.3)	36.3/22	Vascular anomalies 103/191 (53.9%); edematous disorders 88/191 (46.1%)

Data are presented as number (%) unless otherwise indicated.

**Table 2:** Objective outcome: Circumference change at the ankle’s narrowest point in the vascular anomaly group (n = 20).

Parameter	Affected Limb	Unaffected Limb	*p* Value	Effect Size (r)
n	20	20	<0.001	0.83 (large)
Median change (mm)	−2	+0.5		
IQR (mm)	[−4.2, 0.0]	[−0.2, +1.0]		
Statistical test	Wilcoxon signed-rank test (T = 4)			

IQR, interquartile range;. A negative circumference change denotes a decrease (improvement) in limb circumference. Statistical test: Wilcoxon signed-rank test (paired comparison, affected vs. unaffected limb). Effect size r = 0.83 (nonparametric bootstrap 95% CI 0.71–0.89; 10,000 resamples); median paired difference −2 mm (95% CI −3 to −1 mm).

**Table 3:** Subjective outcome: Patient-reported satisfaction score before and after custom-made compression garment use in the edematous disorder group (n = 20).

Parameter	Before	After	*p* Value	Effect Size (r)
n	20	20	<0.001	0.83 (large)
Median score	3	4		
IQR	[2.0, 4.0]	[3.0, 6.0]		
Score improvement, n (%)	17/20 (85.0%)			
Statistical test	Wilcoxon signed-rank test (T = 0)			

IQR, interquartile range;. Statistical test: Wilcoxon signed-rank test (paired comparison, before vs. after garment use). Effect size r = 0.83 (nonparametric bootstrap 95% CI 0.75–0.91; 10,000 resamples); median paired improvement +1 point (95% CI +1 to +2).

## Data Availability

The data presented in this study are available on request from the corresponding author. The data are not publicly available due to privacy and ethical restrictions.
